# Assessment of Validity of Selected Criteria of Fatigue Life Prediction

**DOI:** 10.3390/ma12142310

**Published:** 2019-07-19

**Authors:** Krzysztof Kluger, Roland Pawliczek

**Affiliations:** Department of Mechanics and Machine Design, Opole University of Technology, 45-271 Opole, Poland

**Keywords:** mean stress, fatigue life, multiaxial stress, bending, torsion

## Abstract

The paper reports on the results of a comparison involving mathematical models applied for fatigue life calculations where the mean load value is taken into account. Several models based on the critical plane approach and energy density parameter were tested and analyzed. A fatigue test results for three types of materials are presented in this paper. The specimens were subjected to bending, torsion and a combination of bending with torsion with mean value of the load. Analysis of the calculation results show that the best fatigue life estimations are obtained by using models that are sensitive to the changes of material behavior under fatigue loading in relation to the specified number of cycles of the load.

## 1. Introduction

The phenomenon of material fatigue is a known effect which is of fundamental importance when designing structures working under variable load conditions. Despite the large number of research works, it was not possible to develop a single, universal calculation algorithm that can be used in a wide range of construction materials as well as load conditions of the structure. The formation and accumulation of fatigue damage is a complex phenomenon and depends on many factors, e.g., the type and state of material, geometry of the element, type of loading or stress state [1,2,3].

The fatigue of materials is usually referred to simple stress states, e.g., uniaxial tension-compression, where material characteristics are limited to determining the relationship between the number of cycles to failure and the level of the applied load. In the case of operational loads, the most common is a complex stress state, and in this case, we refer to the results of calculations to uniaxial characteristics using the appropriate hypotheses (calculation models) [1,4,5,6,7].

Among the large number of fatigue hypotheses and computational models, it is difficult to find one universal that would contain a wide range of factors describing the fatigue cracks creation. Moreover, neither of them can offer a comprehensive tool that makes them adequate to use in any kind of the material, geometry and load conditions. The literature in the area contains a variety of fatigue criteria. They are based on various assumptions and parameters describing the process of fatigue. One can distinguish criteria based on stresses, strains and the so-called energy, taking into account both, the state of stress and strain.

In the case of a complex stress state, such parameters as the stress amplitude and the mean stress value cause a change in the orientation of the principal stresses, so their impact on the fatigue life is difficult to predict. The mean value of stress, different from zero, often comes as a result of the own weight of the operating element or of the entire structure, and can arise from preloading of carriers (e.g., V-belts in the gear units). Technological aspects of manufacture of constructions, technologies of joining elements (e.g., welding, explosive welding) are a source of residual stresses, which introduce significant mean loads to the structure. In the situation when the object does not transfer loads with the mean value of the load, omitting the residual stress can introduce a significant error in estimating fatigue life [8,9,10].

The aim of this paper is comparison of the efficiency for selected and most frequently used fatigue life estimation criteria taking into account the effect of the mean stress in multiaxial loading conditions: Gerber [11], Findley [12], Dang Van [13], Carpinteri-Spagnoli [3], Smith-Watson-Topper (SWT) parameter [14], and the stress models by: Kluger-Łagoda [15,16], which were modified with the use of two-parameter fatigue characteristics describing the relation between the values of amplitude and mean stress of Pawliczek-Gasiak model [17]. The paper presents experimental results with calculations for the analyzed models.

## 2. Analyzed Fatigue Models

### 2.1. Models Based on Haigh Diagram

Models described below are given among the group of models that apply mathematical description of Haigh diagram of stress amplitude change σ_a_ (τ_a_) and mean stress value σ_m_ (τ_m_). Despite the fact that some of these criteria use parameters not related to material behavior under fatigue load conditions, they are often used due to the simplicity of computational algorithms and are the basis for reference and verification of the efficiency of more advanced computational models.

By adapting the assumption that the maximum stress, which can be carried out by a material in a single loading state is the stress, which defines the ultimate tensile strength σ_U_, Goodman [18,19] proposed a linear description of the dependence between the stress amplitude change and the mean stress value using the formula:

(1) $$\frac{\sigma_{\mathrm{aeq}}}{\sigma_{\mathrm{eq}}}+\frac{\sigma_{\mathrm{meq}}}{\sigma_{U}}=1$$

The study by Soderberg [20] suggested that for materials in elastic-plastic state, a practical criterion defining material failure is the state during which the yield strength σ_y_ is exceeded. Hence, Equation (1) takes the following form:

(2) $$\frac{\sigma_{\mathrm{aeq}}}{\sigma_{\mathrm{eq}}}+\frac{\sigma_{\mathrm{meq}}}{\sigma_{y}}=1$$

Non-linear properties (σ_a_-σ_m_) (τ_a_-τ_m_) were considered in the model introduced by Gerber [11]:

(3) $$\frac{\sigma_{\mathrm{aeq}}}{\sigma_{\mathrm{eq}}}+{\left(\frac{\sigma_{\mathrm{meq}}}{\sigma_{U}}\right)}^{2}=1$$

As the criterion, the value the equivalent stress σ_eq_ is

(4) $$\sigma_{\mathrm{eq}}=\frac{\sigma_{\mathrm{aeq}}}{1-{\left(\frac{\sigma_{\mathrm{meq}}}{\sigma_{i}}\right)}^{q}}$$

and it is used to calculate the degree of damage based on the fatigue characteristics in simple load conditions (e.g., in a tension-compression test). Exponent q and σ_i_ in Equation (4) adapt the values of: q = 1, σ_i_ = σ_U_ for Goodman’s model, q = 1, σ_i_ = σ_y_ for the normalized Soderberg model, and q = 2, σ_i_ = σ_U_ for the non-linear Gerber equation, respectively.

For all models presented above, stress σ_aeq_ and σ_meq_ are amplitude and mean value of equivalent stress, respectively. The Huber-Mises hypothesis forms the most common one and is used most frequently, as it is able to specify equivalent stress amplitude and mean stress value for multi-axial load for the case of multi-axial fatigue.

As a result of developing and transforming dependencies based on the mean loading value on Haigh diagram (σ_a_-σ_m_ and τ_a_-τ_m_), their limit for stress level corresponding to the unlimited life (fatigue limit) is indicated. It was indicated that material sensitivity on mean loading is not a material constant and depends on the number of the cycles corresponding to the failure of an element. This postulate is the basis for a modification of transforming dependencies for two-parameter fatigue characteristics of metals presented by Gasiak and Pawliczek [21]. The effect of loading conditions and material sensitivity change on mean loading value, together with the number of destructive cycles for SJ355 steel are presented in [17]. The proposed model for calculation of the equivalent amplitude of normal stresses σ_aeq_ has a form:

(5) $$\sigma_{\mathrm{aeq}}=\sqrt{\sigma_{\mathrm{aT}}^{2}+k\tau_{\mathrm{aT}}^{2}}$$

where: k = 3 according to the Huber-Mises hypothesis or k = σ_af_/τ_af_, while σ_aT_, τ_aT_—the amplitude of the corresponding normal and shear stress components is calculated as follows:

(6) $$\sigma_{a_{T}}=\sigma_{a}+\psi_{\sigma }(N)\cdot \sigma_{m}$$

(7) $$\tau_{a_{T}}=\tau_{a}+\psi_{\tau }(N)\cdot \tau_{m}$$

The material sensitivity factor for the asymmetry of cycle ψ_σ_ ψ_τ_ is applied in Equations (6) and (7), and its value is derived for normal and shear stresses. When determining the ψ factor, the change in its value is considered by considering the number of cycles equal to N. Changes in material sensitivity on the asymmetry of cycles can be determined by the experimental dependency [17]:

(8) $$\psi =\eta \cdot N^{\lambda }$$

The approximate values of η and λ coefficients for structural steel for a limited lifetime can be calculated as:

(9) $$\lambda =-0.588\cdot \log\left(\frac{10^{4.7a+b}-1}{10^{6.4a+b}-1}\right)$$

(10) $$\eta =10^{4.7(a-\lambda )+b}-10^{-4.7\lambda }$$

where: $a=\frac{1}{A_{w}}-\frac{1}{A_{j}}$, $b=\frac{B_{j}}{A_{j}}-\frac{B_{w}}{A_{w}}$, while A_j_, B_j_, A_w_, B_w_ are the coefficients of regression line for limited lifetime derived on the basis of fatigue test results for fully reversed loading and repeated—one direction loading. We can note that for complex stress state, these parameters should be designated, both for normal and shear stresses. After determining the amplitudes transformed as in Equations (6) and (7), equivalent stresses should be designated according to the adapted hypothesis on material effort.

### 2.2. Models Based on Critical Plane Approach

The following models belong to the group of models based on stresses and are applied to consider the aspect of critical plane [7]. It is defined as the one, in which maximum failure occurs and the fatigue life depends on a combination of stresses and/or strains acting along a plane. Depending on the state of stresses, environment, geometry of the element and on the amplitude of stresses, the fatigue process is dominated by the formation of cracks in the plane of maximal shear stress or normal stress. Criteria based on the analysis of stress state in the critical plane are considered to refer to factors that destroy material, as they relate to planes in which fatigue cracks initiate and develop. It is believed that they are the closest to fatigue processes occurring in materials. These criteria belong to the group of the most widely developed computational models and have received numerous modifications.

#### 2.2.1. Findley’s Model

Findley proposed the model [12] considering the effect of mean value of stress, where the equivalent amplitude of shear stress on the critical plane (shear plane in this case) takes the form:

(11) $$\tau_{\mathrm{aeq}}=\tau_{n,a}+k\sigma_{n,\max}$$

Weight factor k is specifying the effect of normal stress, and according to Findley, it depends on the number of cycles to failure, and maximal stress on critical plane is σ_n,max_ = σ_n,m_ + σ_n,a_. Findley assumed that the principal directions for proportional loading do not change. The k parameter is determined by fatigue limits in alternating torsion τ_af_ and fully reversed bending σ_af_ by solving the following equation:

(12) $$\frac{\sigma_{\mathrm{af}}}{\tau_{\mathrm{af}}}=\frac{2\sqrt{1+k^{2}}}{\sqrt{1+k^{2}}+k}$$

#### 2.2.2. Dang Van’s Fatigue Model

The Dang Van fatigue criterion [13] is distinguished by mesoscopic (grain boundary) stress observation scale. The Dang Van criterion assumes that fatigue of a material does not occur, if all grains reach a stable state of elastic deformations. It means that after the initial period of loading (after several cycles), the material goes into isotropic hardening and further relationship between stress and strain will occur in the elastic state.

The condition of exceeding the critical deformations depends on the value of the mesoscopic shear stresses τ_μ_ and hydrostatic stresses σ_H,max_.

The above-mentioned stresses are combined by a linear function designating equivalent stress:

(13) $$\tau_{\mathrm{aeq}}=\left|\tau_{\mu }\right|+k\sigma_{H,\max}$$

where

(14) $$k=\frac{\tau_{\mathrm{af}}-\frac{\sigma_{\mathrm{af}}}{2}}{\frac{\sigma_{\mathrm{af}}}{3}}$$

The value of maximum mesoscopic shear stress τ_μ_ is calculated in terms of principal stresses, according to the Tresca hypothesis.

#### 2.2.3. Model Based on the Carpinteri-Spagnoli Criterion

Carpinteri and Spagnoli [22] developed a criterion based on the Gough’s empirical criterion, in which the equivalent stress is calculated by the relation:

(15) $$\sigma_{\mathrm{aeq}}=\sqrt{\sigma_{n,\max}^{2}+k^{2}\tau_{n,a}^{2}}$$

and the coefficient k is determined from the expression:

(16) $$k=\frac{\sigma_{\mathrm{af}}}{\tau_{\mathrm{af}}}$$

The critical plane is defined with respect to mean directions ($\hat{1},\;\hat{2},\;\hat{3}$) of principal stresses, determined by the method where weight function is used. The critical plane lies in the plane $\hat{1},\;\hat{3}$ and is rotated by the angle δ around the axis $\hat{2}$. The value of the angle δ depends on the coefficient k as a function.

(17) $$\delta =\frac{3\pi }{8}\left[1-\frac{1}{k^{2}}\right]$$

#### 2.2.4. Model by McDiarmid

The proposed McDiarmid criterion [23,24] for high cycle multiaxial fatigue can be expressed by equivalent stress in the form of

(18) $$\tau_{\mathrm{aeq}}=\left|\tau_{\max}\right|+k\langle \sigma_{n}\rangle$$

Equation (18) implies that the fatigue failure is caused by a linear combination of normal stresses and shear stresses at the critical plane, being defined by the maximum value of shear stresses. The coefficient k considers a different number of normal stresses during the process of determining fatigue life and is presented by the relation

(19) $$k=\frac{\tau_{\mathrm{af}}}{2\sigma_{U}}$$

#### 2.2.5. Model by Papadopoulos

Papadopoulos [25,26] proposed the fatigue criterion, which combines the approach applied used for the group of criteria based on invariants of the stress state, criteria based on stress mean values and criteria using the concept of the critical plane. Fatigue strength is determined upon a linear combination of the maximum hydrostatic stress σ_H,max_ and the amplitude of generalized shear stress $\langle T_{a}\rangle$ being defined on the critical plane, while the equivalent value can be represented by:

(20) $$\tau_{\mathrm{aeq}}=\langle T_{a}\rangle +k\sigma_{H,\max}$$

where:

(21) $$k=3\frac{\tau_{\mathrm{af}}}{\sigma_{\mathrm{af}}}-\frac{1}{2}$$

#### 2.2.6. Model by Matake

On the basis of observations of the fatigue crack propagation directions that overlap the planes of the maximum shear stresses, Matake [27] has a proposed stress criterion, in which the critical plane is the plane of the maximum shear stress. Normal stress σ_n_(t) acting on the plane accelerates the crack initiation process as the linear function of shear stress. The equivalent stress takes the form of

(22) $$\tau_{\mathrm{aeq}}=\tau_{n,a}+k\sigma_{n,a}$$

The material constant k can be specified on the basis of fatigue limits for fully reversed bending and torsion.

(23) $$k=2\frac{\tau_{\mathrm{af}}}{\sigma_{\mathrm{af}}}-1$$

In the case, when several planes reach the same maximum shear stress value, it is assumed that the critical plane is the surface with higher normal stress.

#### 2.2.7. Model by Kluger-Lagoda

Kluger and Łagoda [15] had proposed a criterion to describe the effect of mean stress, the equivalent value of which is presented in the form:

(24) $$\sigma_{\mathrm{aeq}}=B\tau_{n}+K\sigma_{n}=B\left(\tau_{n,a}+\tau_{n,m}\right)+K\left(\sigma_{n,a}+\sigma_{n,m}\right)$$

If an element is under loading by stress amplitude σ_a_ (for bending or tension-compression) and τ_a_ (for torsion), the value of the amplitude of normal stresses in the direction ***n*** can be written as

(25) $$\sigma_{n,a}=\sigma_{a}\cos\alpha^{2}+\tau_{a}\sin2\alpha$$

and the amplitude of shear stresses in the direction ***s*** as:

(26) $$\tau_{n,a}=-0.5\sigma_{a}\sin2\alpha +\tau_{a}\cos2\alpha$$

The mean values of normal stresses in the direction ***n*** can adapt the form of

(27) $$\sigma_{n,m}=\sigma_{m,p}\cos\alpha^{2}+\tau_{m,p}\sin2\alpha$$

whereas, mean values of the mean shear stresses in the direction ***s*** as

(28) $$\tau_{n,m}=-0.5\sigma_{m,p}\sin2\alpha \;+\tau_{m,p}\cos2\alpha$$

where the angle *α* is the critical plane orientation angle.

The mean stresses of σ_m,k_ and τ_m,k_, applying a correction are used to calculate the normal σ_n,m_ and shear τ_ns,m_ components, designated based on the formula:

(29) $$\sigma_{m,p}=k_{\sigma }\sigma_{m}$$

(30) $$\tau_{m,p}=p_{\tau 1}p_{\tau 2}p_{m}$$

Coefficients p_σ_, p_τ1_, p_τ2_ are derived experimentally, based on the analysis of fatigue test results and their values are, respectively [15]:

(31) $$p_{\sigma }=\sqrt{\frac{\sigma_{\max}}{{\sigma^{\prime }}_{f}}}$$

where σ_max_ = σ_a_ + σ_m_,

(32) $$p_{\tau 1}=\frac{\tau_{a}}{\sqrt{2}\tau_{m}+\tau_{a}}$$

(33) $$p_{\tau 2}=1+\frac{\sigma_{m}}{\sigma_{m}+\tau_{m}}$$

Equivalent stress forms a linear combination of nominal and shear stresses. The ratio of particular components of stress state in the fatigue process depends on B and K factors:

(34) $$B=\frac{\sigma_{a}(N_{f})}{\tau_{a}(N_{f})}$$

(35) $$K=2-\frac{\sigma_{a}(N_{f})}{\tau_{a}(N_{f})}$$

Values of σ_a_(N_f_) and τ_a_(N_f_) are derived on the basis of fatigue equations S-N for simple loading states: Tension (bending), shear (torsion), respectively. In this case, it is important to pay attention to the parallelism of characteristics in the entire range of the high-cycle fatigue. It should be noted that for aluminum alloys, a change in slope factor values of characteristics is very common, so this phenomenon may affect the results of calculations [28].

### 2.3. Models Based on Energy Approach

#### Smith-Watson-Topper Model

Smith, Watson and Topper in the study [14] proposed for description of fatigue phenomenon to consider in calculations, both the stresses and strains. The parameter elaborated by the authors is known as the p_SWT_ parameter and in the group of energy models. Socie [29] proposed a modification to the SWT parameter, which subsequently offers the estimation of the multiaxial fatigue life of proportional and non-proportional cyclic loading. The modification is based on the assumption that stresses and strains are calculated as sizes acting perpendicular to the critical plane. The proposed modification is the most common form that is applied for writing the SWT parameter in the critical plane of the maximal range of normal strain Δε_1_ in the form:

(36) $$p_{\mathrm{SWT}}=\sigma_{n,\max}\frac{\Delta \varepsilon_{1}}{2}$$

By adapting the Manson-Coffin dependency as the effort function, fatigue lifetime can be estimated using:

(37) $$p_{\mathrm{SWT}}=\sigma_{n,\max}\frac{\Delta \varepsilon_{1}}{2}=\frac{{\sigma^{\prime }}_{f}{}^{2}}{2}{\left(2N_{f}\right)}^{2b}+{\sigma^{\prime }}_{f}{\varepsilon^{\prime }}_{f}{\left(2N_{f}\right)}^{b+c}$$

## 3. Experimental Study

A fatigue test was performed in specimens made of 2017A-T4 [15] and 6082-T6 [15,30] aluminum alloys and also of S355J0 [31] steel alloy. Strength properties of the analyzed materials are provided in Table 1. For the 2017A-T4 aluminum alloy and S355J0 steel alloy, the tests included bending, torsion conditions and two combinations of constant-amplitude of proportional bending with torsion, for which τ(t) = σ(t) and τ(t) = 0.5σ(t) with zero and non-zero mean value. For the 6082-T6 aluminum alloy, additional combinations of constant-amplitude of bending with torsion, for which τ(t) = 0.25σ(t) were analyzed.

**Figure 1:**
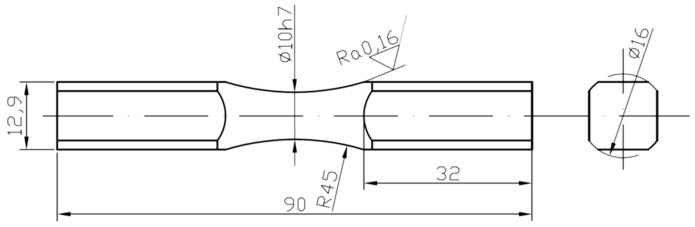
Shape and dimensions (in millimetres) of the specimen of the analyzed materials used in fatigue tests.

The tests projected in this study were performed in room temperature by using the MZGS100 fatigue testing machine that can apply the control of the resultant moment of loading the specimen, on specimens presented in Figure 1. The loads were of a sinusoidal nature with a frequency of about 25–29 Hz. The amplitudes and the mean value of the load were changed according to the test requirements. For each combination of load at least two or three specimens were used. The nominal stress amplitude and nominal mean stress value were used in calculations.

For S355J0 steel, functions for calculations of material sensitivity factor on the asymmetry of cycles for normal and shear stresses were defined experimentally [31]:

—For bending: ψ_σ_(N) = 3.124⋅N^−0.162^,

—For torsion ψ_σ_ (N) = 2.890⋅N^−0.148^.

## 4. Analysis of Results

A wide range of empirical research tests allows for estimation of the effectiveness of fatigue life prediction using the analyzed models regarding the impact of mean loading. The estimation involved the computational models for each material in cases of various loading. The application of various materials for the purpose of this study allowed for performing estimation of fatigue life prediction in selected models in relation to the material grade.

**Figure 2:**
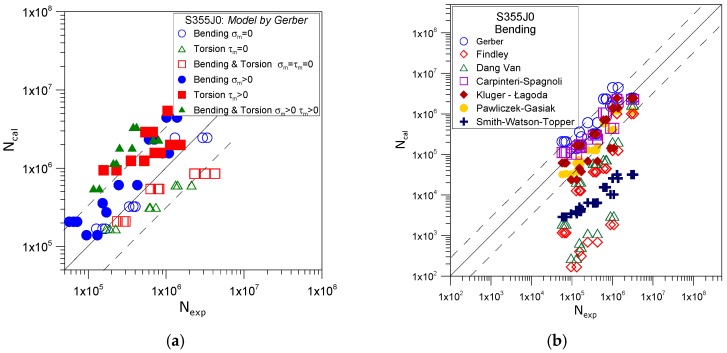
Comparison of calculated and experimental fatigue life for S355J0 steel; (**a**) for the Gerber model and various types of load; (**b**) for bending and various calculation models.

Figure 2 illustrates an exemplary comparison of the calculated fatigue life (N_cal_) and the one derived experimentally (N_exp_) for S355J0 steel under various loading conditions using the Gerber’s model (Figure 2a) and a comparison of the applied computational models according to one type of loading-bending (Figure 2b). The equivalent stress σ_eq_ was determined using the Gerber model (Equation (3)). This stress was used to determine the calculated fatigue life N_cal_ using a standard S-N curve for bending (see Table 1, slope factor and intercept).

**Figure 3:**
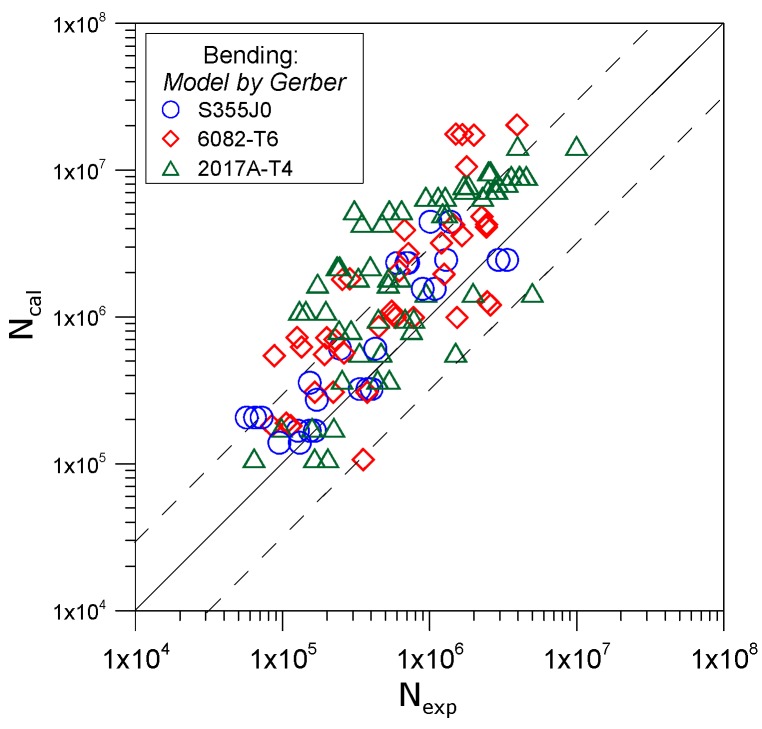
Comparison of calculated and experimental fatigue life by applying Gerber’s model for three tested materials subjected to bending.

Application in the study of different materials also offered the assessment of the efficiency of fatigue life prediction in selected models with respect to the type of the material. Figure 3 presents an exemplary graph comparing the stability of computational results with those obtained experimentally by using Gerber’s model.

The solid line in Figure 2 and Figure 3 represents perfect compatibility between the results derived in calculations and experimental ones. The dotted lines represent the range of scatter of results, for which the ratio of the number of cycles to failure derived by calculations N_cal_ and those derived experimentally N_exp_ corresponds to results the value of 3 and ⅓.

The feasibility of the analyzed computational models and results obtained by an empirical test was estimated by the analysis of computational scatter-band as [1,32]:

(38) $$E_{\mathrm{eq}}=\sqrt{E_{m}^{2}+E_{\mathrm{std}}^{2}}$$

where E_m_ is the mean scatter, while E_std_ is the standard deviation to mean scatter, respectively derived from the relation:

(39) $$E_{m}=\frac{1}{n}\sum_{i=1}^{1}E_{i},$$

(40) $$E_{\mathrm{std}}=\sqrt{\frac{1}{n-1}\sum_{i=1}^{1}{\left(E_{i}-E_{m}\right)}^{2}}$$

where n is the number of specimens. The scatter band for the particular results was determined as

(41) $$E_{i}=\log\frac{N_{i,\mathrm{cal}}}{N_{i,\exp}}$$

**Figure 4:**
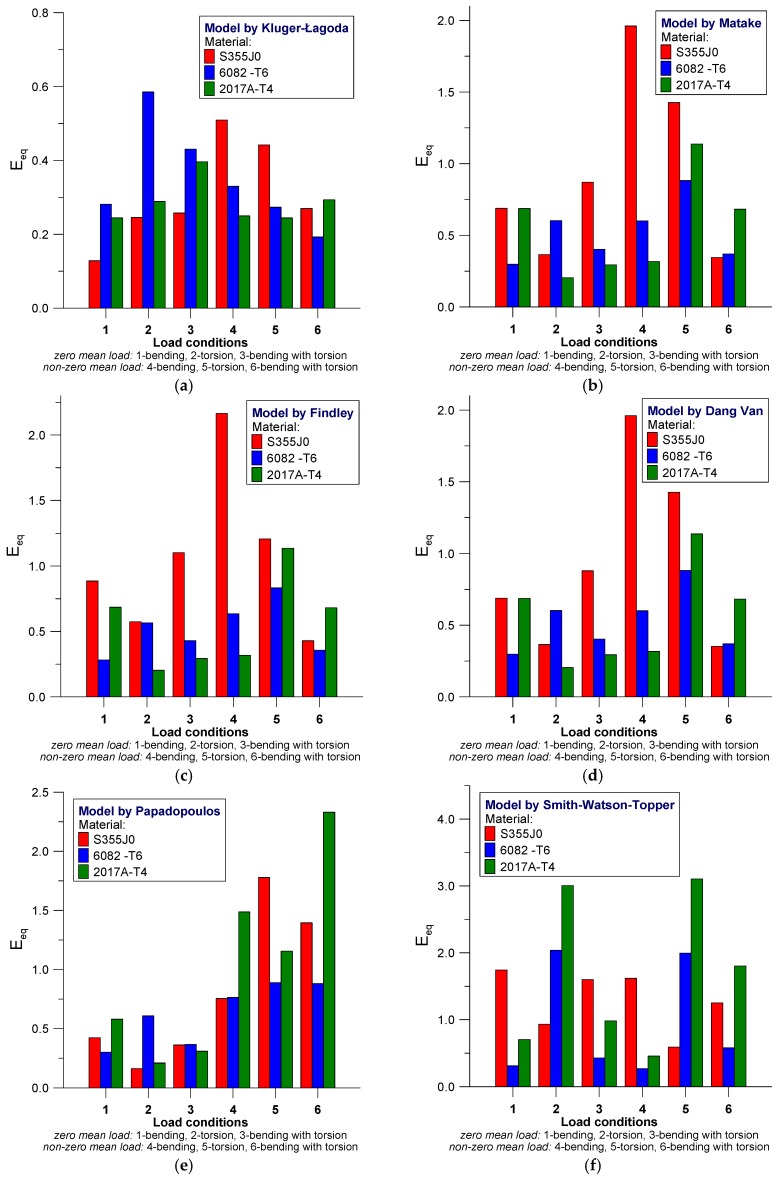
Scatter bands E_eq_ for the analyzed models corresponding to tested materials. (**a**) Kluger-Lagoda; (**b**) Matake; (**c**) Findley; (**d**) Dang Van; (**e**) Papadopoulos; (**f**) Smith-Watson-Topper.

**Figure 5:**
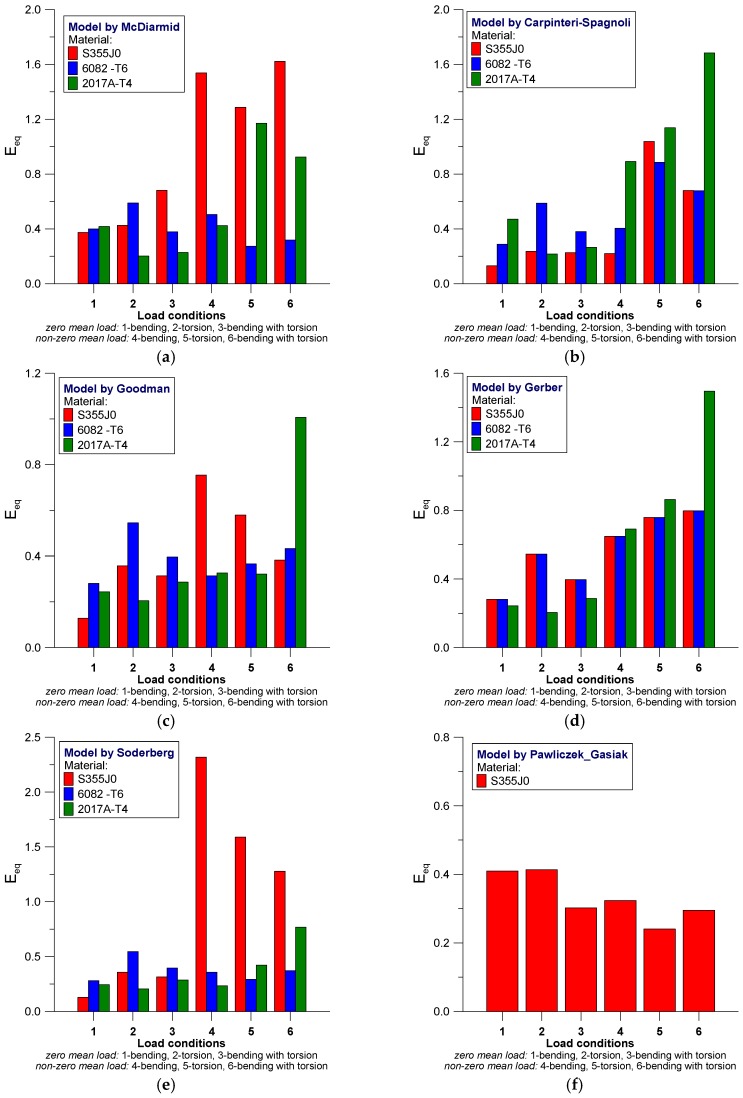
Scatter bands E_eq_ for the analyzed models corresponding to tested materials, cont. (**a**) McDiarmid; (**b**) Carpinteri-Spagnoli; (**c**) Goodman; (**d**) Gerber; (**e**) Soderberg; (**f**) Pawliczek-Gasiak.

The scatter values derived on the basis of Equation (38) for analyzed models according to the materials used in the test are presented in Figure 4 and Figure 5.

In most cases, it can be seen that efficiency in estimating fatigue life depends on the manner of loading and type of material. We can note that the use of Kluger-Lagoda model (Figure 4a) does not lead to scatter bands not exceeding the value of 0.6 and the maximum values are received for torsion in 6082-T6 aluminum alloy and S355J0 steel under bending and torsional loading with mean value of loading. By using the model by Matake (Figure 4b) greater scatters are received for non-zero mean value of loading under bending and torsion in S355J0 steel, wherein, the case of bending relates also to aluminum alloys. For Findley’s model (Figure 4c) the largest scatter bands are derived for S355J0 steel, regardless of the manner of loading, whereas in the case of complex loading with non-zero mean value, satisfactory results of fatigue life estimation were obtained, which were comparable for all analyzed materials. The Dang Van fatigue model (Figure 4d) yields very similar results in terms of the nature and values as they are derived from Findley’s model. Additionally, the fatigue life prediction for S355J0 steel demonstrates large scatters. Models proposed by Papadopoulos (Figure 4e) are characterized by wide-ranging scatter plots of results for non-zero mean value loads in all of the analyzed materials, while loading with zero mean value indicates two to three times lower scatters. The Smith-Watson-Topper model (Figure 4f) demonstrates extended scatters for both tested aluminum alloys under torsion loading with—both for zero as well as the non-zero mean value, where the scatter band receive the values: E_eq_ = 2 and 3 for 6082-T6 and 2017A-T4, respectively. For S355J0 steel the scatter bands are ranging from 0.5 to 1.75.

Figure 5 shows the continuation of the analysis for the remaining models.

Scatter bands of fatigue life estimation results calculated according to the model by McDiarmid is presented in Figure 5a, wherein for loading with mean value, the poor results were obtained for S355J0 steel and 2017A-T4 alloy under torsion and bending with torsion with mean value of loading. Moreover, in the Carpinteri-Spadnoli model (Figure 5b) the poorest estimation results are derived under loading with mean value for bending, torsion and torsion with bending.

Fatigue life estimations based on models by Goodman (Figure 5c) and Gerber (Figure 5d) are characterized by similarity of scatter bands, wherein, the dependence by Gerber seems to be more sensitive to the type of test material—scatter levels for all of the analyzed cases of loading are similar for three of the analyzed materials. For 2017A-T4 alloy in complex stress state with mean value, both of the analyzed dependencies indicate approximately two times wide-ranging scatter bands than the other cases of loading. As a result of the application of the model proposed by Soderberg (Figure 5e), three to four times larger scatters with regard to the estimation results for S355J0 steel, being tested under loading with mean stress values. For S355J0 steel the fatigue life prediction was additionally performed using the model proposed by Pawliczek and Gasiak (Figure 5f). This model is characterized by a scatter plot on the results that ranged from 0.25 to 0.4 for all the analyzed cases of loading with both, the zero and non-zero mean values of stress.

The above observations indicate that most of these models demonstrate high sensitivity to loading conditions. In certain cases, there can be seen larger discrepancies between computational results and experimental results under loading with non-zero mean values.

**Figure 6:**
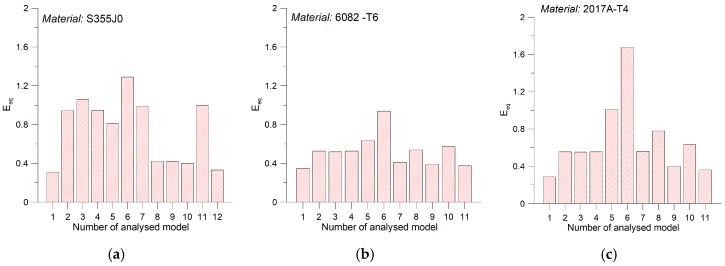
Average scatter bands E_eq_ for materials (**a**) S355J0; (**b**) 6082-T6; (**c**) 2117A-T4 for the analyzed models: 1. Kluger-Łagoda; 2. Matake; 3. Findley; 4. Dang Van; 5. Papadopoulos; 6. Smith-Watson-Topper; 7. McDiarmid; 8. Carpinteri-Spagnoli; 9. Goodman; 10. Gerber; 11. Soderberg; 12. Pawliczek-Gasiak.

In order to assess the flexibility of the analyzed calculation models considering the effect of mean load and the type of loading (i.e., simple or complex), mean value of scatter bands E_eq_ were subsequently derived for each of the materials (Figure 6). For S355J0 steel the most adequate averaged results were obtained for models by Kluger-Lagoda, Carpinteri-Spagnoli, Goodman, Gerber and Pawliczek-Gasiak, where scatter band of the results was E_eq_ < 0.4. In the case of aluminum alloys, the discrepancy was not equally evident in terms of the analyzed calculation models, however, the most efficient results of computations were obtained for the Kluger-Lagoda dependency, and then for Goodman and Soderberg’s models.

## 5. Conclusions and Finding

The study reported in this paper was concerned with the discussion of the results of a fatigue test performed for three materials: S355J0 steel alloy and two aluminum alloys designated as: 6082-T6 and 2017A-T4. Specimens were tested under bending, torsion, and various combinations of bending with torsion. To estimate fatigue life under multiaxial loading including the influence of the mean value, a several, well known-models for High diagram σ_a_-σ_m_ (τ_a_-τ_m_) description were used. Models based on the critical plane, where the maximum stresses as the sum of the amplitude of stress and the mean value have been investigated. The paper also presents the model taking into account changes of the sensitivity of the material to mean loads depending on the number of cycles to failure. The scatter-band of results expressed as the relationship between the fatigue lifetime based on calculations N_cal_ and the fatigue life based on empirical tests N_exp_ were also applied as the criteria for estimating the suitability of the models.

Results from the analysis results in the statement of the following conclusions:

For S355J0 steel the most accurate mean conformity of the calculated and experimental results was obtained for models by Kluger-Lagoda, Carpinteri-Spagnoli, Goodman, Gerber and Pawliczek-Gasiak;
For both aluminum alloys, the models proposed by Kluger-Lagoda and also by Goodman and Soderberg proved to be most adequate, other models indicate higher discrepancies for cases of loading with load mean values;
In the case of aluminum alloys, the Smith-Watson-Topper model demonstrates higher sensitivity in specimens subjected to torsional loading;
Models in which the mean load is considered as a total of stress amplitude and mean stress (maximum stress as a criterion value) produce highly deteriorated fatigue life estimation results;
For the analyzed loading conditions, the most accurate fatigue life estimations are obtained by using models that consider the material sensitivity change as the load mean value varies according to the number of cycles to failure (Pawliczek-Gasiak) and those taking into account the relationship between basic fatigue characteristics for bending and torsion (Kluger-Lagoda).

## Figures and Tables

**Table 1:** Strength and fatigue properties of the analyzed materials.

Properties	Designation and Unit	2017A-T4	6082-T6	S355J0
Young’s modulus	E, [GPa]	72	72	213
Tensile strength limit	σ_u_, [MPa]	545	385	611
Yield strength limit	σ_y_, [MPa]	395	365	394
Fatigue strength coefficient	σ′_f_, [MPa]	643	651	880
Slope factor of S-N curves (for bending)	m_σ_	7.03	8.00	7.10
Intercept of S-N curves (for bending)	A_σ_	21.87	23.83	23.80
Slope factor of S-N curves (for torsion)	m_τ_	6.87	7.7	11.7
Intercept of S-N curves (for torsion)	A_τ_	19.94	21.4	32.8
Fatigue limit for bending	σ_af_, [MPa]	142	126	271
Fatigue limit for torsion	τ_af_, [MPa]	78	74	175

## Nomenclature

b	fatigue strength exponent
B, K	weight factors depending on the number of cycles to failure
c	fatigue ductility exponent
E_eq_	computational scatter-band
E_i_	scatter-band for the individual results
E_m_	the mean scatter-band
E_std_	the standard deviation to mean scatter-band
k	weight factor
N_f_	number of cycles to failure
N_i,cal_	calculated fatigue life for individual specimens
N_i,exp_	experimental fatigue life for individual specimens
p_SWT_	Smith-Watson-Topper parameter
p_σ_, p_τ1_, p_τ2_	coefficients derived experimentally
$\langle T_{a}\rangle$	amplitude of generalized shear stress
α	critical plane orientation angle
δ	angle of rotation of the principal stress directions
Δε_1_	normal strain range on the critical plane
ε′_f_	fatigue ductility coefficient
η, λ	factors determined based on the Haigh diagram for normal and shear stresses, respectively
σ′_f_	fatigue strength coefficient
σ_a_	amplitude of normal stress
σ_aeq_	amplitude of equivalent normal stress calculated for multiaxial stress state
σ_af_	fatigue limits for fully reversed bending
σ_aT_	transformed normal stress amplitude due to the mean value
σ_eq_	amplitude of equivalent stress for fully reversed uniaxial stress
σ_H,max_	hydrostatic stresses
σ_m_	mean value of normal stress
σ_meq_	mean value of equivalent stress calculated for multiaxial stress state
σ_n_	the tensile normal stress on the plane of maximum shear stress
σ_n,a_	normal stress amplitude on critical plane
σ_n,m_	normal mean stress on critical plane
σ_n,max_	maximal, normal stress on critical plane
σ_U_	ultimate tensile strength
σ_y_	yield strength
τ_af_	fatigue limit for alternating torsion
τ_μ_	mesoscopic shear stresses
ψ_σ_ ψ_τ_	material sensitivity factor for the asymmetry of cycle
τ_a_	amplitude of shear stress
τ_aeq_	amplitude of equivalent shear stress calculated for multiaxial stress state
τ_aT_	transformed shear stress amplitude due to the mean value
τ_m_	mean value of shear stress
τ_max_	the maximal shear stress
τ_n_	shear stress on critical plane
τ_n,a_	shear stress amplitude on the critical plane
τ_n,m_	shear mean stress on the critical plane
